# Forecasting the Incidence of Mumps Based on the Baidu Index and Environmental Data in Yunnan, China: Deep Learning Model Study

**DOI:** 10.2196/66072

**Published:** 2025-02-06

**Authors:** Xin Xiong, Linghui Xiang, Litao Chang, Irene XY Wu, Shuzhen Deng

**Affiliations:** 1 Department of Epidemiology and Health Statistics Xiangya School of Public Health Central South University Changsha China; 2 Department of School Health Yunnan Provincial Center for Disease Control and Prevention Kunming China; 3 Hunan Provincial Key Laboratory of Clinical Epidemiology Central South University Changsha China

**Keywords:** mumps, deep learning, baidu index, forecasting, incidence prediction, time series analysis, Yunnan, China

## Abstract

**Background:**

Mumps is a viral respiratory disease characterized by facial swelling and transmitted through respiratory secretions. Despite the availability of an effective vaccine, mumps outbreaks have reemerged globally, including in China, where it remains a significant public health issue. In Yunnan province, China, the incidence of mumps has fluctuated markedly and is higher than that in mainland China, underscoring the need for improved outbreak prediction methods. Traditional surveillance methods, however, may not be sufficient for timely and accurate outbreak prediction.

**Objective:**

Our study aims to leverage the Baidu search index, representing search volumes from China’s most popular search engine, along with environmental data to develop a predictive model for mumps incidence in Yunnan province.

**Methods:**

We analyzed mumps incidence in Yunnan Province from 2014 to 2023, and used time series data, including mumps incidence, Baidu search index, and environmental factors, from 2016 to 2023, to develop predictive models based on long short-term memory networks. Feature selection was conducted using Pearson correlation analysis, and lag correlations were explored through a distributed nonlinear lag model (DNLM). We constructed four models with different combinations of predictors: (1) model BE, combining the Baidu index and environmental factors data; (2) model IB, combining mumps incidence and Baidu index data; (3) model IE, combining mumps incidence and environmental factors; and (4) model IBE, integrating all 3 data sources.

**Results:**

The incidence of mumps in Yunnan showed significant variability, peaking at 37.5 per 100,000 population in 2019. From 2014 to 2023, the proportion of female patients ranged from 41.3% in 2015 to 45.7% in 2020, consistently lower than that of male patients. After excluding variables with a Pearson correlation coefficient of <0.10 or *P* values of <.05, we included 3 Baidu index search term groups (disease name, symptoms, and treatment) and 6 environmental factors (maximum temperature, minimum temperature, sulfur dioxide, carbon monoxide, particulate matter with a diameter of 2.5 µm or less, and particulate matter with a diameter of 10 µm or less) for model development. DNLM analysis revealed that the relative risks consistently increased with rising Baidu index values, while nonlinear associations between temperature and mumps incidence were observed. Among the 4 models, model IBE exhibited the best performance, achieving the coefficient of determination of 0.72, with mean absolute error, mean absolute percentage error, and root-mean-square error values of 0.33, 15.9%, and 0.43, respectively, in the test set.

**Conclusions:**

Our study developed model IBE to predict the incidence of mumps in Yunnan province, offering a potential tool for early detection of mumps outbreaks. The performance of model IBE underscores the potential of integrating search engine data and environmental factors to enhance mumps incidence forecasting. This approach offers a promising tool for improving public health surveillance and enabling rapid responses to mumps outbreaks.

## Introduction

Mumps is an acute respiratory infectious disease caused by the mumps virus, characterized by unilateral or bilateral facial swelling [1]. It is transmitted through contact with the respiratory secretions of infected individuals, with an incubation period typically ranging from 16 to 18 days [2]. Nonspecific symptoms such as fever, headache, and malaise could occur before the inflammation of the parotid and other salivary glands [1,2]. While mild symptoms are more common, severe complications such as aseptic meningitis, encephalitis, sensorineural deafness, and orchitis can also develop [3,4]. Out of 2 doses of the mumps vaccine have shown nearly 90% effectiveness in protecting against mumps [5], reducing the global incidence of mumps by 97% [6] Until 2019, a total of 122 countries have introduced mumps vaccination [7], and the number reached to 124 Member States by the end of 2023 [8]. However, in recent years, mumps outbreaks have reemerged in many countries, even those with high vaccine coverage, such as the United States [9], the United Kingdom [10], and Ireland [11], which may have many potential reasons, but the waning immunity may contribute majorly [12].

In China, mumps is classified as a Class C notifiable infectious disease. Despite being vaccine-preventable, mumps remained the most prevalent disease among the 44 notifiable infectious diseases from 2008 to 2017 in persons aged 6 to 22 years, with incidences of 83.207 per 100,000 population in 2008 and 69.771 per 100,000 population in 2017 [13]. Similar to the global condition, even after mumps was included in the expanded immunization program in mainland China in 2008, an abnormal increase in incidence was observed after the program [13,14]. Although no exact reason, waning immunity, changing environment, high population mobility, along with misinformation about the vaccination may play roles [7,15,16]. After the onset of the COVID-19 pandemic, the incidence of mumps in mainland China was 7.37 and 6.48 per 100,000 population in 2022 and 2023, ranking fourth and fifth among Class C notifiable infectious diseases, respectively [17,18]. These trends highlight the continued public health challenge posed by mumps in China.

Yunnan, a province located in southwestern China, has experienced marked variability in mumps incidence. The average annual percentage change in incidence was 13.97% from 2004 to 2018, ranking third, just slightly lower than that of Hunan (15.15%) and Hainan (15.10%) provinces [19]. Furthermore, the mumps incidence in 2022 and 2023 was 14.2 and 12.8 per 100,000 population, respectively, both of which were notably higher than the national averages. These sharp fluctuations in the incidence of mumps, along with relatively higher incidence presented challenges in predicting mumps outbreaks, particularly given the lag of traditional surveillance system, which rely on case reports from medical and health institutions.

Several studies have developed prediction models for the incidence of mumps in China [20,21]. However, due to regional variations of mumps incidence, these models might not be suitable for Yunnan province. In addition, these studies only used mumps incidence, environmental factors data or both as predictors, which may not be able to explain the nonseasonal fluctuations. Incorporating alternative predictors, such as search engine query data, may improve the timeliness and accuracy of outbreak predictions. Previous studies have identified search engine data, such as that from Twitter [rename as X], Google, and Baidu, along with environmental factors, as effective predictors of certain infectious diseases such as influenza and chicken pox, due to their lower cost and higher timeliness [22-24]. Baidu, the most popular search engine in China, provides a rich source of real-time data that can be leveraged for predictive purposes. Long short-term memory (LSTM) networks, introduced by Hochreiter and Schmidhuber [25] in 1997, are a specialized type of recurrent neural network designed to capture long-term dependencies in sequential data by mitigating the vanishing and exploding gradient problems [25]. These networks have shown exceptional performance in capturing temporal dependencies in larger horizons [26]. Therefore, our study aims to use multisource data to develop a prediction model of mumps incidence in Yunnan province based on LSTM.

## Methods

### Data Sources

Our study used a total of 3 time series data about Yunnan province, including the incidence of mumps, the Baidu index [27], and environmental factors.

We obtained the daily number of mumps cases with their age, sex, and careers, and the annual population data from the Yunnan Center for Disease Control and Prevention, which collected accurate case characteristics from the Chinese information system for infectious diseases control and prevention, for the period between January 1, 2014, and December 31, 2023. From the system, we collected a total of 20 career types in our study, reporting the number of patients of the top 6 most common ones while grouping the remaining careers under the category “other careers.” In addition, since more than 80% of patients were younger than 20 years, we organized the age distribution into 5-year groups for those younger than 20 years, while others were presented as a separate category. The Baidu index [27] is the weighted sum of the search frequencies of various search terms by scientifically analyzing and calculating based on users’ search volumes on Baidu, which is the most popular search engine in China. By extensive search, we initially collected daily Baidu index for 30 search terms in Yunnan from January 1, 2016, and December 31, 2023. After group discussion, we excluded 3 search terms due to irrelevance and another 3 because they were introduced after 2016, resulting in incomplete data (Table S1 in Multimedia Appendix 1). Ultimately, we included 24 search terms in our study.

We collected information about environmental factors during January 1, 2016, and December 31, 2023, from China Meteorological Administration [28], including maximum temperature (°C), minimum temperature (°C), air quality index, particulate matter (PM) with an aerodynamic diameter less than or equal to 2.5 μm (PM_2.5_; measured as μg/m^3^), PM with an aerodynamic diameter less than or equal to 10 μm (PM_10_; measured as μg/m^3^), sulfur dioxide (SO_2_; measured as μg/m^3^), nitrogen dioxide (NO_2_; measured as μg/m^3^), carbon monoxide (CO; measured as mg/m^3^), and ozone (O_3_; measured as μg/m^3^).

### Statistical Analysis

#### Feature Selection and Descriptive Analysis

To predict the weekly incidence of mumps, we calculated the weekly values for each variable. The weekly mumps incidence and Baidu index [27] were obtained by summing their daily values, while the weekly environmental factors were calculated as the mean values for each week. We performed Pearson correlation tests to determine the correlation between each variable and the incidence of mumps. After excluding variables with a Pearson correlation coefficient (PCC) of <0.10 or a *P* value of <.05, we consequently included 16 search terms and 6 environmental factors (Table S2 in Multimedia Appendix 1). These search terms were classified into 3 groups based on their content: disease name, symptoms, and treatment. We then calculated the weekly sum of the Baidu index for these 3 groups as the predictors.

Our study used the Kolmogorov-Smirnov test to inspect the normality of continuous variables. Normally distributed continuous variables were described as mean and SD, while nonnormally distributed continuous variables are described as median and IQR.

#### Log Correlation Analysis

Previous studies have demonstrated that environmental factors and the Baidu index [27] had a lag impact on the incidence of infectious disease, which means exposure affects health for a period of days [22,29]. To capture these complex relationships, we used the distributed lag nonlinear model (DLNM). DLNM is a modeling framework that simultaneously represents nonlinear exposure-response relationships and lagged effects using cross-basis functions [30]. We used natural spline functions for both the exposure-response and lag-response components, allowing for flexible modeling of nonlinear associations. We selected DLNM over other nonlinear methods, such as generalized additive models, because DLNM is specifically tailored to handle both nonlinear and lagged effects, which is crucial for understanding the temporal dynamics of environmental influences on mumps incidence. Our study used R package DLNM (version 2.4.7) to make DLNM between the Baidu index [27] and environmental factors and mumps incidence [31].

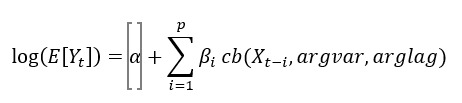



Yt represents the outcome value (mumps incidence) at week t while E[Yt] is its expected value. α refers to the interception term. p refers to the maximum lag time, which was 3 weeks in our study considering the incubation and previous study [32]. βi the regression coefficient for the i-th lag time, representing the effect size of the exposure variable at lag i on the outcome variable. cb(Xt - i, argvar, arglag) is a cross-basis function used to capture the nonlinear relationship between the exposure variable Xt-i and the lag time. This function depends on 3 parameters: Xt-i represents the value of the exposure variable at time t-i, argvar were parameters defining the effect of the exposure variable, and arglag were parameters defining the lag effect. Relative risks (RR) were used to assess the effect of a specific exposure level at one lag time compared with the reference exposure value.

#### Model Construction and Performance Evaluation

**Figure 1:**
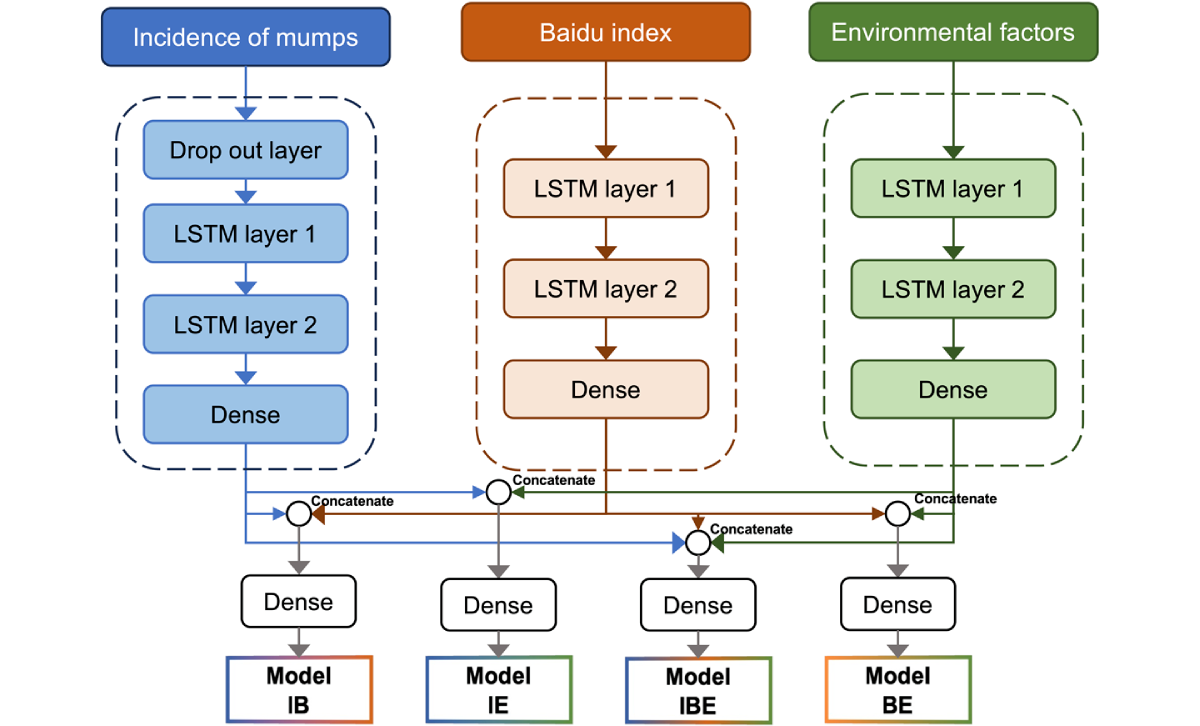
Model constructions. LSTM: Long short-term memory. Model BE: model including Baidu index and environmental factors; Model IB: model including mumps incidence and Baidu index; Model IE: model including mumps incidence and environmental factors; Model IBE: model including mumps incidence and Baidu index and environmental factors.

Our study developed 4 models based on LSTM to predict mumps incidence 1 week in advance (Figure 1). First, to better capture the characteristics of the 3 time series data, we constructed 3 primary LSTM models for each data set. The primary model for mumps incidence included a dropout layer to prevent overfitting, followed by 2 LSTM layers and a dense layer. The Baidu index [27] and environmental factors models each consisted of 2 LSTM layers followed by a dense layer. Among the 3 models, we used data from the previous 2 weeks to predict the incidence of mumps one week in advance. Subsequently, we concatenated the outputs of these primary models to construct composite models. Model IB combined the mumps incidence and Baidu index outputs, model IE combined the mumps incidence and environmental factors outputs, model BE combined the Baidu index [27] and environmental factors outputs, and model IBE combined the outputs from all 3 primary models. Each composite model then passed the concatenated outputs through an additional dense layer to produce the final prediction. This approach ensured that primary models effectively integrated information from different data sources for accurate predictions. To simulate real-world scenarios where future data are unknown and must be predicted based on past observations, we divided our data sets into train, validation and test set based on time. The train set interval was from January 1, 2016, to December 31, 2021. Data from January 1, 2022, to December 31, 2022, and those from January 1, 2023, to December 31, 2023, were used as the validation and test sets, respectively. These models were constructed by keras3 package [ONEIROS] (version 1.0.0), tensorflow package [Google Brain Team] (version 2.16.0) in R (version 4.4.1; R Core Team).

Out of 4 performance metrics were applied to evaluate these 4 models: coefficient of determination (*R*^2^), mean absolute error (MAE), mean absolute percentage error (MAPE), and root-mean-square error (RMSE). *R*^2^ refers to the proportion of the variance for a dependent variable that is explained by the model, with values less than 1, where larger values indicate better performance. MAE, MAPE, and RMSE measure the errors between predictions and actual values, with MAE and RMSE ranging greater than 0, and MAPE ranging from 0 to 100%, where lower values indicate better model accuracy. Compared with the MAE, MAPE represents the average of absolute percentage errors expressed as a percentage, making it more suitable for comparison across different data sets and models, while RMSE is more sensitive to outliers and larger errors.

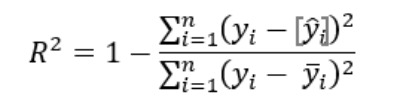

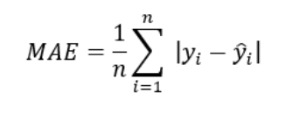

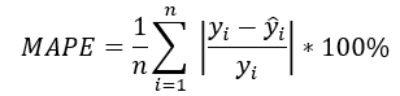

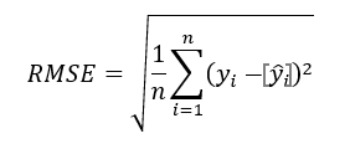









In these equations, n was the number of observations, while *y_i_* and *y_i_* refer to the actual incidence of mumps in the *i_th_* week and its prediction, respectively.

### Ethical Considerations

Our research was approved by the Medical Ethics Committee, Xiangya School of Public Health, Central South University, Changsha, China (XYGW-2024-65). Informed consent was waived by our institutional review board because all data were fully anonymized before being accessed by the researchers, ensuring that no identifiable information about individuals was available. Therefore, compensation is not applicable in our study.

## Results

### The Temporal Distribution of Mumps Incidence and Predictors

**Figure 2:**
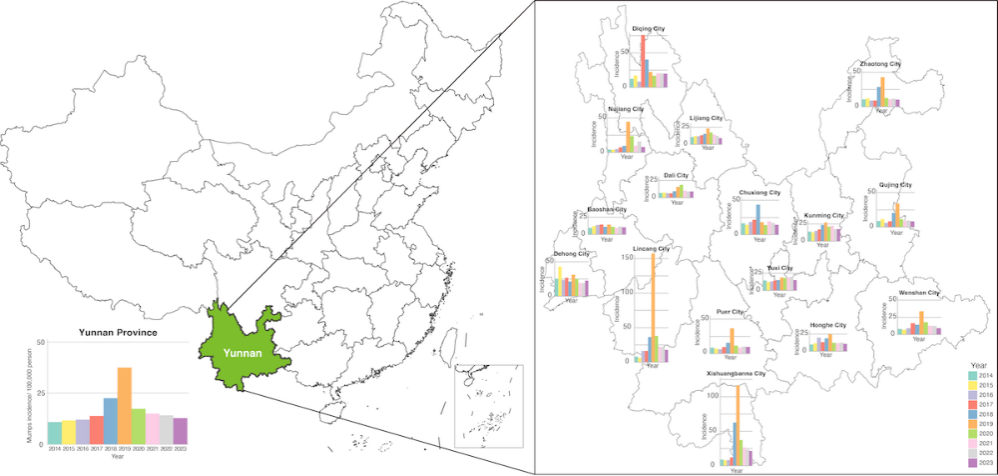
Annual incidence of mumps (per 100,000 population) in Yunnan province from 2014 to 2023.

In Yunnan Province, the incidence of mumps increased from 10.9 per 100,000 population in 2014, peaked at 37.5 per 100,000 population in 2019, and then steadily decreased to 12.8 per 100,000 population by 2023 (Figure 2). The outbreaks of mumps occurred from 2017 to 2020 in different cities in Yunnan. From 2014 to 2023, the proportion of female patients remained fairly stable, ranging from 41.3% in 2015 to 45.7% in 2020. (Table 1). The median age of patients showed some fluctuations over the years, starting at 7 years (IQR 4-12) in 2014, peaking at 11 years (IQR 7-14) in 2019, and then decreasing to 6 years (IQR 4-10) by 2023. The proportion of cases in children between 5 and 10 years old remained substantial, with the highest proportion recorded in 2023 (43.7%). Students consistently represented the largest affected group, comprising 44.3% to 67.5% of cases in 2014 and 2019, respectively.

**Table 1:** Characteristics of reported mumps patients in Yunnan from 2014 to 2023.

		2014 (N=5343)	2015 (N=5611)	2016 (N=5850)	2017 (N=6843)	2018 (N=11,249)	2019 (N=18,703)	2020 (N=8592)	2021 (N=7296)	2022 (N=6788)	2023 (N=6085)
Female		2290 (42.9)	2320 (41.3)	2484 (42.5)	2927 (42.8)	4961 (44.1)	8214 (43.9)	3929 (45.7)	3250 (44.5)	2929 (43.1)	2599 (42.7)
Age (years), median (IQR)		7 (4-12)	8 (5-11)	8 (5-12)	9 (5-13)	10 (6-14)	11 (7-14)	8 (5-14)	6 (4-11)	6 (4-11)	6 (4-10)
**Age groups (years), n (%)**											
	<5	1389 (26)	1387 (24.7)	1456 (24.9)	1329 (19.4)	1679 (14.9)	2266 (12.1)	2051 (23.9)	2183 (29.9)	1940 (28.6)	1700 (27.9)
	5 to <10	2082 (39)	2224 (39.6)	2300 (39.3)	2484 (36.3)	3492 (31)	5236 (28.0)	2801 (32.6)	2828 (38.8)	2873 (42.3)	2661 (43.7)
	10 to <15	1007 (18.8)	1105 (19.7)	1074 (18.4)	1807 (26.4)	3786 (33.7)	7019 (37.5)	1618 (18.8)	944 (12.9)	850 (12.5)	715 (11.8)
	15 to <20	162 (3)	200 (3.6)	210 (3.6)	308 (4.5)	1098 (9.8)	2272 (12.1)	679 (7.9)	289 (4.0)	201 (3.0)	152 (2.5)
	≥20	703 (13.2)	695 (12.4)	810 (13.8)	915 (13.4)	1194 (10.6)	1910 (10.2)	1443 (16.8)	1052 (14.4)	924 (13.6)	857 (14.1)
**Careers, n (%)**											
	Student	2368 (44.3)	2608 (46.5)	2654 (45.4)	3627 (53)	7027 (62.5)	12618 (67.5)	3912 (45.5)	2794 (38.3)	2571 (37.9)	2269 (37.3)
	Diaspora children	1246 (23.3)	1229 (21.9)	1073 (18.3)	1001 (14.6)	1169 (10.4)	1660 (8.9)	1259 (14.7)	1059 (14.5)	972 (14.3)	796 (13.1)
	Nursery children	992 (18.6)	1041 (18.6)	1271 (21.7)	1267 (18.5)	1798 (16.0)	2302 (12.3)	1961 (22.8)	2398 (32.9)	2326 (34.3)	2163 (35.5)
	Peasant	519 (9.7)	539 (9.6)	614 (10.5)	670 (9.8)	926 (8.2)	1546 (8.3)	1100 (12.8)	761 (10.4)	666 (9.8)	616 (10.1)
	Unemployment	33 (0.6)	33 (0.6)	45 (0.8)	48 (0.7)	57 (0.5)	101 (0.5)	62 (0.7)	55 (0.8)	61 (0.9)	68 (1.1)
	Teacher	17 (0.3)	20 (0.4)	24 (0.4)	29 (0.4)	56 (0.5)	100 (0.5)	62 (0.7)	32 (0.4)	30 (0.4)	19 (0.3)
	Other careers	168 (3.1)	141 (2.5)	169 (2.9)	201 (2.9)	216 (1.9)	376 (2)	236 (2.7)	197 (2.7)	162 (2.4)	154 (2.5)

**Figure 3:**
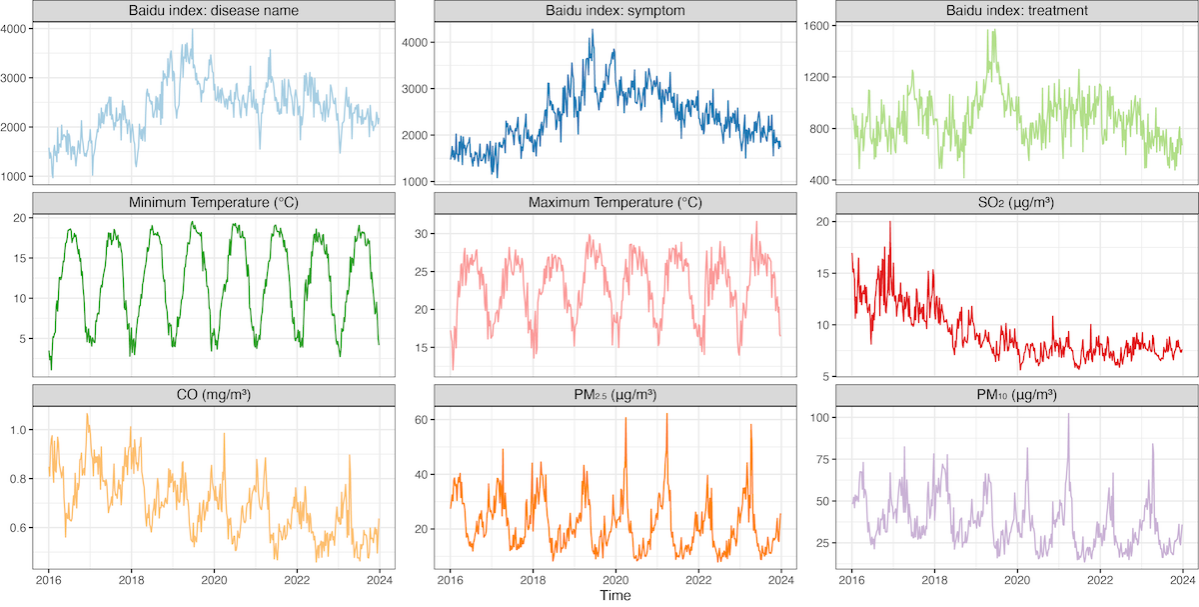
The temporal distribution of weekly Baidu index and environmental factors in Yunnan Province from 2016 to 2023 CO: carbon monoxide; SO2: sulfur dioxide; PM: particulate matter; PM2.5: particulate matter with a diameter of 2.5 µm or less; PM10: particulate matter with a diameter of 10 µm or less.

The temporal distribution from 2016 to 2023 revealed distinct trends in both the Baidu search index and environmental factors (Figure 3). The distribution of the 3 Baidu index mirrored the pattern observed in mumps incidence, with a peak in 2019 and followed by a decline. Most environmental factors except SO₂ displayed obvious seasonal cycles. Maximum and minimum temperatures reached peaks in summer, while air pollutants, including CO, PM_2.5,_ and PM_10_, had higher concentrations in winter. In addition, SO₂ and CO showed a long-term decreasing trend over the 10-year period.

### Correlation Between Predictors and Incidence of Mumps

The Pearson correlation analysis reveals that 3 Baidu search terms, disease name (r=0.67), symptom (r=0.66), and treatment (r=0.53), show strong positive correlations with mumps incidence in Yunnan Province from 2016 to 2023 (Table 2). Among environmental factors, both minimum (r=0.17) and maximum temperature (r=0.16) have weak positive correlations, while pollutants including SO₂ (r=–0.16), CO (r=–0.12), PM_2.5_ (r=–0.20) and PM_10_ (r=–0.10) show weak negative correlations with mumps incidence.

**Table 2:** Distribution of predictors and Pearson correlation coefficient between weekly predictors and mumps incidence in Yunnan province from 2016 to 2023.

Predictors		Baidu search terms in Chinese			Median (IQR)		PCC^a^		*P* value
**Baidu search terms**									
	Disease name	—			2394 (2053-2697)		0.67		<.001
	Mumps	流行性腮腺炎			560 (457-655)		0.42		<.001
	Parotitis	腮腺炎			1397 (1225-1564)		0.76		<.001
	Zha sai (a colloquial name for mumps in Chinese)	痄腮			301 (174-418)		0.36		<.001
	San xian yan (a term phonetically like “parotitis” in Chinese)	三线炎			114 (57-172)		0.39		<.001
	Symptom	—			2360 (1928-2821)		0.66		<.001
	Jaw pain	下巴疼			114 (57-174)		0.38		<.001
	Swelling on one side of the face	半边脸肿			0 (0-57)		0.23		<.001
	Pain at the base of the ear	耳根痛			0 (0-0)		0.16		<.001
	Facial swelling	脸肿			440 (348-524)		0.39		<.001
	Complications of parotitis	腮腺炎并发症			0 (0-57)		0.24		<.001
	Early symptoms of parotitis	腮腺炎的早期症状			115 (57-294)		0.26		<.001
	Symptoms of parotitis + parotitis symptoms	腮腺炎的症状+腮腺炎症状			1315 (876-1560)		0.33		<.001
	Parotitis with orchitis	腮腺炎睾丸炎			0 (0-0)		0.13		.01
	Is parotitis contagious?	腮腺炎传染吗			436 (172-508)		0.48		<.001
	Treatment	—			860 (729-1000)		0.53		<.001
	What medicine to take for parotitis	腮腺炎吃什么药			464 (441-506)		0.30		<.001
	How to treat parotitis	腮腺炎怎么治疗			300 (233-405)		0.54		<.001
	Treatment of parotitis	腮腺炎的治疗			57 (0-115)		0.13		.007
**Environmental factors**									
	Maximum temperature, °C		—	24 (20.2-26.3)		0.16		<.001	
	Minimum temperature, °C		—	12.8 (7.6-17.2)		0.17		<.001	
	SO^b^_2_ (μg/m^3^)		—	8.2 (7.4-10.6)		–0.20		<.001	
	CO^c^ (mg/m^3^)		—	0.7 (0.6-0.8)		–0.12		.02	
	PM^d^_2.5_ (μg/m^3^)		—	19.5 (13.8-27.4)		–0.20		<.001	
	PM_10_ (μg/m^3^)			34.3 (25.5-46.0)		–0.10		.04	

^a^PCC: Pearson correlation coefficient.

^b^SO: sulfur dioxide.

^c^CO: carbon monoxide.

^d^PM: particulate matter.

**Figure 4:**
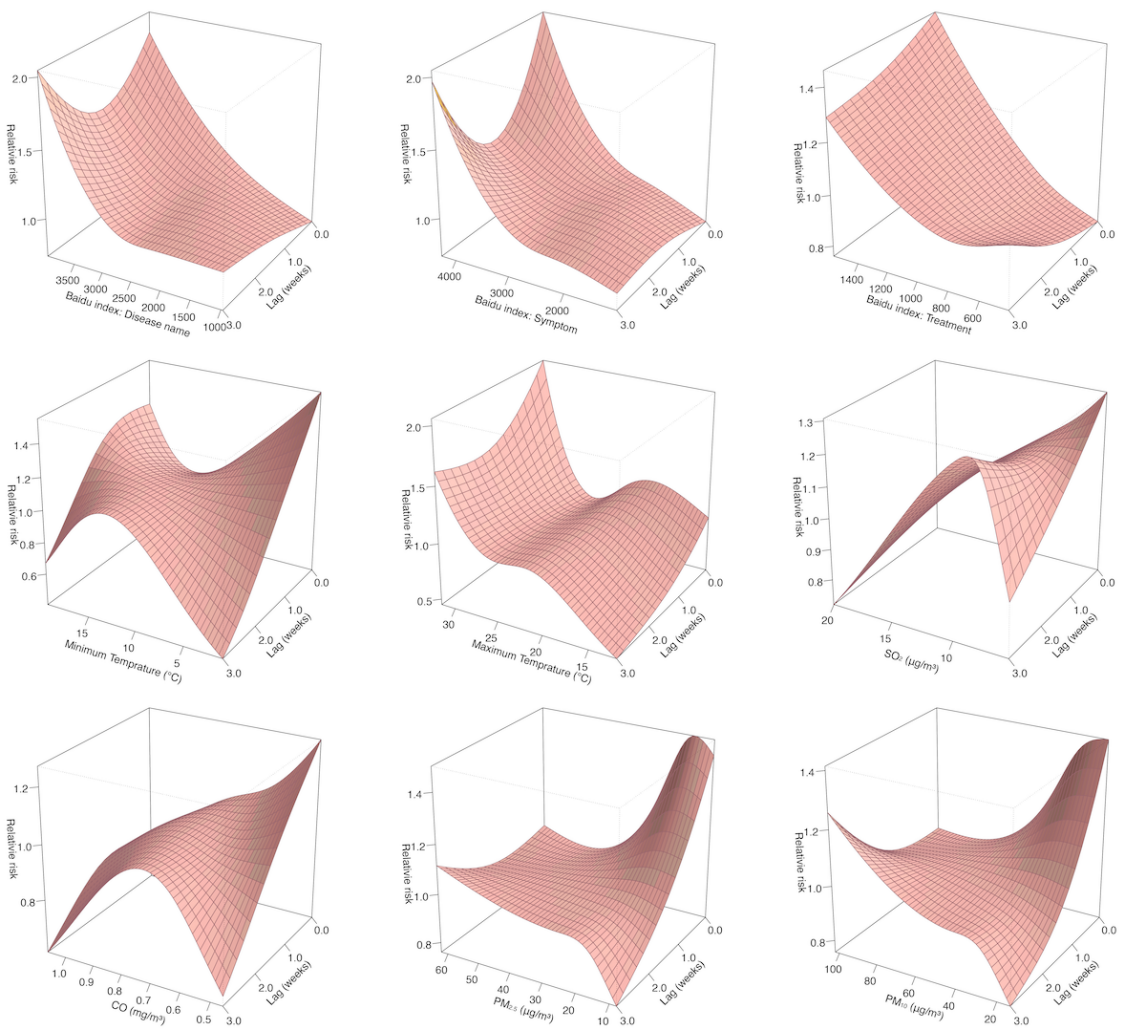
Relative risks for mumps incidence by predictors and lags using distributed lag nonlinear models in Yunnan province from 2016 to 2023, with references at median of each predictor.

Considering the potential lag impact of these predictors on mumps incidence, we leveraged DLNM to explore their short-term impacts (Figure 4). The impact of various predictors on the incidence of mumps varies across different lag weeks. For 3 Baidu search terms, the RR consistently increases with the rising Baidu index. The analysis also revealed nonlinear associations between temperatures and incidence, with minimum temperature showing a U-shaped curve and maximum temperature exhibiting a complex pattern. In addition, elevated levels of SO_2_, CO, PM_2.5_, and PM_10_ generally corresponded with decreases in RR at a 1-week lag.

### Model Performances and Comparisons

**Figure 5:**
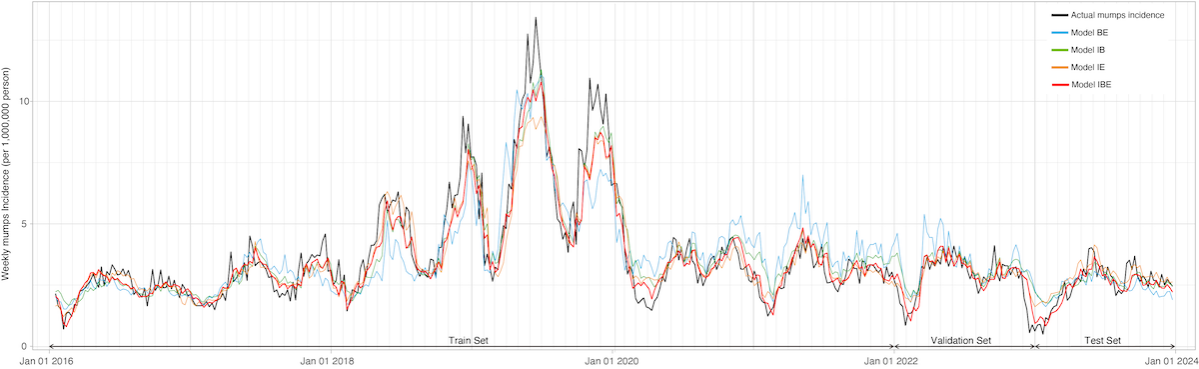
Predictions of weekly mumps incidence 1 week in advance by different models in Yunnan province from 2016 to 2023. Model BE: a model including Baidu index and environmental factors; model IB: the model including mumps incidence and Baidu index; model IE: a model including mumps incidence and environmental factors; model IBE: the model including mumps incidence, Baidu index, and environmental factors.

Table 3 shows the comparisons of the performance metrics of 4 models with different combinations of predictors. Model IBE, which includes all 3 predictors, consistently outperformed the other models in both the train and validation set, as well as the test set. Specifically, model IBE achieved the highest *R*² values (0.89 in the train and validation set, 0.72 in the test set) and the lowest error metrics, with an MAE of 0.51, MAPE of 15.2%, and RMSE of 0.74 in the train and validation set, and an MAE of 0.33, MAPE of 15.9%, and RMSE of 0.43 in the test set. In comparison, the IE model (*R*²=0.54, MAE=0.45, MAPE=28.2%, and RMSE=0.55) and the IB model (*R*²=0.49, MAE=0.45, MAPE=29.2%, and RMSE=0.58) showed similar levels of prediction accuracy in the test set, both outperforming model BE, which had the worst performance (*R*²=0.33, MAE=0.54, MAPE=32.0%, and RMSE=0.66). The actual values and model predictions of the weekly incidence of mumps are presented in Figure 5.

**Table 3:** Performance metrics of different models.

		Model BE^a^	Model IE^b^	Model IB^c^	Model IBE^d^
**Train and validation set**					
	*R* ^2^	0.80	0.84	0.84	0.89
	MAE^e^	0.69	0.59	0.65	0.51
	MAPE^f^ (%)	21.2	17.1	21.6	15.2
	RMSE^g^	0.97	0.87	0.88	0.74
**Test set**					
	*R* ^2^	0.33	0.54	0.49	0.72
	MAE	0.54	0.45	0.45	0.33
	MAPE (%)	32.0	28.2	29.2	15.9
	RMSE	0.66	0.55	0.58	0.43

^a^Model BE: the model included Baidu index and environmental factors.

^b^Model IB: the model included mumps incidence and Baidu index.

^c^Model IE: the model included mumps incidence and environmental factors.

^d^Model IBE: the model included mumps incidence, Baidu index, and environmental factors.

^e^MAE: mean absolute error.

^f^MAPE: mean absolute percentage error.

^g^RMSE: root-mean-square error.

## Discussion

### Principal Findings

Our study presented the incidence of mumps from 2014 to 2023 in Yunnan, a representative province of irregular mumps activity, and found continuous mumps outbreaks in different cities from 2017 to 2020. Using multisource data, we constructed models for predicting the incidence of mumps one week in advance based on LSTM. Model IBE (ie, the model including the incidence of mumps, Baidu index, and environmental factors) showed the best performance among 4 models.

### Comparison to Previous Work

The mean incidence of mumps in Yunnan from 2014 to 2023 was 16.9 per 100,000 persons, which is lower than that of the period between 2004 to 2012 (26.2/100,000) in Yunnan and comparable to the incidence in mainland China from 2013 to 2018 (16.9/100,000) [19]. The 10-year incidence pattern of mumps in Yunnan shows an increase followed by a decrease starting in 2020. The decline may be related to the COVID-19 pandemic, which extensively reduced opportunities for interpersonal contact, thereby blocking the spread of infectious diseases. However, we do not have a definite explanation for the peak in 2019. Given the rapidly increasing number of cases among students in 2019, factors such as vaccine coverage, waning immunity, and the close gathering of adolescents on campuses may have contributed to the outbreak [3,33].

Previous studies have found nonlinear associations between meteorological factors and the incidence of several infectious diseases including mumps [34,35], influenza [23,24,36], and chickenpox [37]. These findings suggest meteorological factors are common predictors for the field of infectious disease prediction. Our study found 6 environmental factors with a PCC of >0.10 and presented their lag correlations with the incidence of mumps. The most relevant factors were PM_2.5_ and SO_2_, both showing weak negative correlations (PCC=–0.2) with mumps incidence in Yunnan. Notably, the PCC is obviously lower than that of other infectious disease. For example, a study in Singapore found a stronger correlation (PCC=0.32) between minimum temperature and the COVID-19 pandemic incidence [38]. This variability suggests that while environmental factors can be valuable for predicting some infectious diseases, they may not be sufficient for accurate prediction across all diseases, highlighting the need for additional predictors.

In recent years, using search engine data, such as Google Trends [39], and the Baidu index [24,40], to predict the incidence of infectious disease has become popular and demonstrated excellent predictive performance. The advantages of search engine data included timeliness, low cost, and sometimes stronger correlation with disease incidence than other data sources. Given the different disease outcomes, fairly comparing our study with other research using search engine data is challenging, as this is, to our knowledge, the first study to construct a mumps prediction model using search engine data. However, we noted that Yang et al [24] and Choi and Ahn [39], who predicted influenza incidence in mainland China and South Korea respectively, achieved similar or even better performance using Baidu index and Google Trends alone, compared with our study’s combination of the Baidu index and environmental factors. It suggests that the contribution of search engine data to incidence prediction may vary depending on types of disease, regions, and data sources. In our study, the PCCs of the Baidu index of 3 search term groups were higher than those of environmental factors. This may be because the Baidu index, derived from individuals’ active search activities, better reflected the incidence peak between 2018 to 2020. In contrast, the relatively stable environmental factors might contribute more to the seasonal trends in incidence but failed to capture the sharp fluctuations observed during outbreaks.

Our study deployed multisource data to construct and compare 4 models, with the model IBE demonstrating superior performance. Notably, when Baidu index data was incorporated, model IBE exhibited even greater predictive accuracy compared with the IE model. This demonstrates the capability of the Baidu index to predict mumps incidence and the potential of integrating multiple data sources to complement the surveillance systems in predicting mumps more accurately, as these data sets can complement one another and provide a more comprehensive understanding of the influencing factors and early signs of disease spread. Further research could build on the proposed framework in our study or explore the integration of additional data sources, such as social media activity and movement behaviors, to further enhance the predictive models.

### Strengths and Limitations

To our knowledge, our study is the first to develop a prediction model for the incidence of mumps that combines the Baidu index and environmental factors as predictors, and it is also the first to create such a model specifically for Yunnan province, China. In addition, by leveraging data such as the Baidu index, our model offers a novel and practical approach to monitor mumps incidence 1 week in advance.

However, there are several limitations to our study. First, at the city level rather than the provincial level, we observed different outbreak times among cities from 2017 to 2020, which may indicate the spread trajectory of mumps and be important for its control and prevention. Unfortunately, the time-series forecast model in our study cannot leverage this geographical information. Future studies could explore integrating temporal and spatial data, which may be crucial for preventing widespread transmission. Second, some Baidu search terms were introduced after 2016 (eg, the search term “Symptoms and treatment of mump” was introduced on April 2, 2018), thereby decreasing the number of available predictors of the Baidu index, which may compromise the performance of Baidu index. Nonetheless, after extensive research, our study identified and included a sufficient number of predictors. Third, we did not have access to some meteorological factors such as sunshine duration and wind speed, which have been identified as effective predictors in other studies, potentially hindering better performance of our models. Future research should incorporate a broader range of meteorological variables to improve prediction accuracy further. Fourth, applying the model to practical public health interventions still faced several challenges. Future work should focus on developing a user-friendly tool that can automatically collect predictors and output results, making the model more accessible and actionable for public health authorities. Finally, due to economic and geographic variations in China [19], various epidemic patterns of infectious diseases, and the different nature of data sets from other search platforms, this model may not generalize well across different regions, diseases, or data sources without careful adaptation and validation. While the model in this study was applied to mumps, similar approaches could potentially be applied to other infectious diseases with seasonal or outbreak-driven incidence patterns. Diseases such as influenza, chickenpox, and hand, foot, and mouth disease, where public awareness, mobility, and environmental factors play a role in transmission, might benefit from similar predictive frameworks. Future studies should focus on tailoring models to specific diseases and data sets to evaluate their broader applicability.

### Conclusions

In conclusion, our study showed the incidence of mumps in Yunnan province, China, from 2014 to 2023, highlighting a sharp peak around 2019. We used data from 2016 to 2023, including mumps incidence, the Baidu index, and environmental factors, to construct 4 prediction models based on LSTM networks. Among these models, the model IBE demonstrates the best performance. This indicates the feasibility of using search engine data and environmental factors to forecast the incidence of mumps, offering a valuable tool for public health surveillance and rapid outbreak assessment.

## Appendix

Multimedia Appendix 1 Additional tables.

## Abbreviations

CO: carbon monoxide
DLNM: distributed nonlinear lag model
LSTM: long short-term memory
MAE: mean absolute error
MAPE: mean absolute percentage error
PCC: Pearson correlation coefficient
PM: particulate matter
PM2.5: particulate matter with a diameter of 2.5 µm or less
PM10: particulate matter with a diameter of 10 µm or less
RMSE: root-mean-square error
RR: relative risk
SO2: sulfur dioxide
