# Supplementary material for: Forecasting the Incidence of Mumps Based on the Baidu Index and Environmental Data in Yunnan, China: Deep Learning Model Study
Source: J Med Internet Res. 2025 Feb 6;27:e66072. doi: 10.2196/66072 (PMC11843052; doi:10.2196/66072)
Supplement: Multimedia Appendix 1 [file jmir_v27i1e66072_app1.docx]

**Supplementary**

[Table S1. Six excluded Baidu search terms in group discussion. 2](#_Toc178538660)

[Table S2. Characteristics, Pearson correlation coefficient and selection result of potential predictors. 3](#_Toc178538661)

# Table S1. Six excluded Baidu search terms in group discussion.

| **Baidu search terms** | **Baidu search terms in Chinese** | **Exclusion** | **Reason of exclusion** |
| --- | --- | --- | --- |
| Parotid tumor | 腮腺肿瘤 | √ | Irrelevance |
| Parotid gland | 腮腺 | √ | Lack of relevance |
| PowerPoint of mumps | 流行性腮腺炎PPT | √ | Irrelevance |
| Symptoms and treatment of mump | 腮腺炎的症状和治疗 | √ | Introduced on April 2, 2018 |
| What medication for mumps gets better fast | 腮腺炎吃什么药好得快 | √ | Introduced on April 11, 2022 |
| Measles, Mumps, and Rubella vaccine | 麻腮风疫苗 | √ | Introduced on July 12, 2021 |

# Table S2. Characteristics, Pearson correlation coefficient and selection result of potential predictors.

| **Variables** | **Baidu search terms in Chinese** | **Median [IQR]** | ***r*** | ***P* value** | **Inclusion** |
| --- | --- | --- | --- | --- | --- |
| **Environmental factors** | - |  |  |  |  |
| Air quality index | - | 38.0 [29.7; 49.1] | -0.08 | .08 | × |
| CO | - | 0.7 [0.6; 0.8] | -0.12 | .02 | **√** |
| Maximum temperature | - | 24.0 [20.2; 26.3] | 0.17 | <.001 | **√** |
| Minimum temperature | - | 12.8 [7.6; 17.2] | 0.16 | <.001 | **√** |
| NO_2_ | - | 14.3 [11.8; 17.3] | 0.00 | .95 | × |
| O_3_ | - | 49.9 [42.2; 70.7] | -0.07 | .13 | × |
| PM_10_ | - | 34.3 [25.5; 46.0] | -0.10 | .04 | **√** |
| PM_2.5_ | - | 19.5 [13.8; 27.4] | -0.20 | <.001 | **√** |
| SO_2_ | - | 8.2 [7.4; 10.6] | -0.20 | <.001 | **√** |
| **Baidu search term** |  |  |  |  |  |
| Mumps | 流行性腮腺炎 | 560.0 [457.0; 655.0] | 0.42 | <.001 | **√** |
| Parotitis | 腮腺炎 | 1397.0 [1225.0; 1564.0] | 0.76 | <.001 | **√** |
| Zha sai (a colloquial name for mumps) | 痄腮 | 301.0 [174.0; 418.0] | 0.36 | <.001 | **√** |
| San xian yan (a term phonetically like ‘parotitis’ in Chinese) | 三线炎 | 114.0 [57.0; 172.0] | 0.39 | <.001 | **√** |
| Jaw pain | 下巴疼 | 114.0 [57.0; 174.0] | 0.38 | <.001 | **√** |
| Swelling on one side of the face | 半边脸肿 | 0.0 [0.0; 57.0] | 0.23 | <.001 | **√** |
| Pain at the base of the ear | 耳根痛 | 0.0 [0.0; 0.0] | 0.16 | .001 | **√** |
| Facial swelling | 脸肿 | 440.0 [348.0; 524.0] | 0.39 | <.001 | **√** |
| Complications of parotitis | 腮腺炎并发症 | 0.0 [0.0; 57.0] | 0.24 | <.001 | **√** |
| Early symptoms of parotitis | 腮腺炎的早期症状 | 115.0 [57.0; 294.0] | 0.26 | <.001 | **√** |
| Symptoms of parotitis + parotitis symptoms | 腮腺炎的症状+腮腺炎症状 | 1315.0 [876.0; 1560.0] | 0.33 | <.001 | **√** |
| Parotitis with orchitis | 腮腺炎睾丸炎 | 0.0 [0.0; 0.0] | 0.13 | <.001 | **√** |
| Is parotitis contagious? | 腮腺炎传染吗 | 436.0 [172.0; 508.0] | 0.48 | <.001 | **√** |
| Symptoms of zha sai | 痄腮的症状 | 57.0 [0.0; 58.0] | 0.01 | .81 | × |
| Symptoms of mumps | 流行性腮腺炎症状 | 0.0 [0.0; 0.0] | 0.03 | .55 | × |
| Mumps vaccine | 流行性腮腺炎疫苗 | 0.0 [0.0; 0.0] | 0.04 | .38 | × |
| Treatment of mumps | 流行性腮腺炎的治疗 | 0.0 [0.0; 0.0] | 0.00 | .94 | × |
| Prevention of mumps | 流行性腮腺炎的预防 | 0.0 [0.0; 0.0] | 0.01 | .92 | × |
| Home remedies of mumps | 腮腺炎偏方 | 0.0 [0.0; 0.0] | 0.10 | .04 | × |
| What medicine to take for parotitis | 腮腺炎吃什么药 | 464.0 [441.0; 506.0] | 0.30 | <.001 | **√** |
| How to treat parotitis | 腮腺炎怎么治疗 | 300.0 [233.0; 405.0] | 0.54 | <.001 | **√** |
| Treatment of parotitis | 腮腺炎的治疗 | 57.0 [0.0; 115.0] | 0.13 | .008 | **√** |
| Treatment methods of parotitis | 腮腺炎的治疗方法 | 0.0 [0.0; 0.0] | -0.08 | .09 | × |
| Measles, Mumps, Rubella vaccine+ Measles, Mumps, Rubella | 麻风腮疫苗+麻风腮 | 57.0 [0.0; 58.0] | 0.01 | .91 | × |

IQR, interquartile range; *r*: Pearson correlation coefficient.
